# A cross-sectional analysis of all vena cava filter placement over 9-years in Brazil: trends and mortality rates in a population of over 200 million

**DOI:** 10.1016/j.clinsp.2026.100874

**Published:** 2026-02-20

**Authors:** Clara Sanches Bueno, Hedra Marques Santos, Júlia Freire Castanheira, Bruno Jeronimo Ponte, Felipe Soares Oliveira Portela, Marcelo Fiorelli Alexandrino da Silva, Marcelo Passos Teivelis, Alexandre Fioranelli, Nelson Wolosker

**Affiliations:** a Faculdade Israelita de Ciências da Saúde Albert Einstein, São Paulo, SP, Brazil; b Hospital Israelita Albert Einstein, São Paulo, SP, Brazil; c Faculdade de Ciências Médicas da Santa Casa de São Paulo, São Paulo, SP, Brazil; d Universidade de São Paulo, São Paulo, SP, Brazil

**Keywords:** Venous thromboembolism, Vena cava filters, Healthcare disparities, Brazil, Unified health system, Heath administration

## Abstract

**Background:**

Venous Thromboembolism (VTE) is a major global health concern, with Vena Cava Filters (VCFs) serving as an alternative to anticoagulation in high-risk patients. Brazil’s VCF trends remain understudied, particularly in its dual public-private healthcare system. This database study allows a broad overview of the Brazilian healthcare landscape.

**Methods:**

This cross-sectional nationwide analysis used Brazilian public (SUS/TabNet) and private (ANS/D-TISS) healthcare databases (2015–2023) to assess VCF placement rates, regional disparities, and in-hospital mortality. Data included 21,630 procedures, analyzed via generalized linear models (Gamma/Poisson distributions).

**Results:**

The private system performed 57% of VCFs (12,323) despite covering only 25% of the population, with a 4.25-fold higher procedure rate per capita than the public system (29.45 vs. 6.42 per-million). Women accounted for 57.8% of recipients. The Southeast region dominated (70% private, 40.5% public), while the North and Midwest had the lowest rates. Mortality rates were comparable (public 6.7% vs. private 7.4%, *p* = 0.066), peaking during COVID-19. Procedure volumes rose 140% over 9-years, contrasting with declining U.S. trends post-FDA restrictions.

**Conclusions:**

Brazil’s VCF use reflects systemic disparities, with private-system overrepresentation and regional gaps. This study enables unprecedented insights into large-scale VCF implementation across diverse healthcare subsystems.

## Highlights

• The private sector performs the majority of procedures despite serving a minority of the population.
• National procedure volume more than doubled (140% increase) in almost a decade.
• The Southeast region is responsible for 70% of all private procedures.

## Introduction

Venous Thromboembolism (VTE), which includes Deep Vein Thrombosis (DVT) and Pulmonary Embolism (PE),^1^ represents a significant global health burden, with an estimated 5% lifetime prevalence in adult populations.^2^ The rising incidence of VTE reflects demographic and epidemiological transitions, including aging populations, increasing prevalence of obesity, sedentary lifestyles, and enhanced diagnostic sensitivity through advanced imaging techniques and biomarker assays.^3^^,^^4^ Anticoagulation therapy remains the gold standard of VTE treatment. However, Vena Cava Filters (VCF) are an important alternative for specific scenarios, such as when a patient has acute VTE and an absolute contraindication to anticoagulation or when recurrent PE occurs despite adequate anticoagulant therapy.^5^, ^6^, ^7^

Although the placement of VCFs is a minimally invasive, percutaneous procedure, it is not risk-free. Rare but potentially serious complications can occur, such as filter migration, venous wall penetration, and adjacent organ involvement (e.g., duodenal perforation).^8^ Historically, there has been a trend in the United States toward the overuse of VCFs, with studies indicating that over 50% of procedures were for questionable indications in the early 2000s.^9^ In contrast, studies from the same period in Brazil did not show this trend; VCF placement there was more selective and based on well-established criteria.^10^

Brazil's universal healthcare system, known as *Sistema Único de Saúde* (SUS), is a publicly funded program designed to provide full-spectrum medical care to all citizens.^11^ Although the constitution guarantees free healthcare access to all citizens, around 25% of the population opts for additional private health services (Private), often funded through employer-sponsored plans. As a result, approximately 75% of Brazilians rely exclusively on the public healthcare system.^12^

The Public system maintains a national anonymized database that tracks surgical procedures, including all VCF implantations performed in public hospitals. Recently, data on procedures performed within the Private has become publicly available, covering interventions from 2015 to 2023. This new information allows for comparative studies between public and private healthcare systems and provides a broad overview of the Brazilian healthcare landscape. The analysis does not include only out-of-pocket payment data, which represent a minority of expenditures (less than 5%).^13^ Consequently, it enables a thorough assessment of the utilization of VCF in Brazil.

Notably, no population-level study on raw data (as opposed to estimation-based) has yet evaluated VCF placement across an entire nation. Globally, the most extensive analysis of VCF use comes from the U.S. Nationwide Inpatient Sample (NIS), which provides estimates based on a stratified 20% sample of discharges from hospitals participating in the Healthcare Cost and Utilization Project (HCUP).^14^^,^^15^ In Brazil, while some studies have examined VCF placement using SUS databases, none have encompassed both Public and Private datasets, leaving a gap in understanding the full scope of procedural trends, disparities, and outcomes.^10^^,^^16^

Our study represents a significant advancement by offering the first complete national assessment of VCF utilization patterns, covering all 26 states of this continental-scale nation. Our analysis uniquely incorporates data from both the Public and Private. This approach enables unprecedented insights into large-scale VCF implementation across diverse healthcare subsystems.^17^

## Objectives

To study all cases requiring VCF placement in Brazil, within the Public and Private, from 2015 to 2023, using a big data system. The authors intended to analyze variations in the number of procedures over time, trends by geographic region, patient characteristics, and in-hospital mortality rates.

### Materials and methods

This retrospective population-based study analyzed publicly available data from the TabNet platform, of the Department of Informatics of the Unified Health System (DATASUS) and from the Private (D-TISS). TabNet provides open-access data on procedures performed in the Public system, which accredited hospitals must report for reimbursement. Similarly, the platform (D-TISS), managed by the National Supplementary Health Agency (ANS), provides open-access procedure data for the Private health system, excluding out-of-pocket payments.

This research was deemed exempt by the institutional ethics committee and was institutionally registered under the number 10337 (*Sistema Gerenciador de Projetos de Pesquisa*, SGPP). All data provided by TabNet and D-TISS are anonymous and publicly available. For this reason, the Institutional Review Board (*Conselho de Revisão Institucional*) waived the requirement for informed consent forms. This study was conducted in accordance with the STROBE statement.^18^

Data on VCF placement were collected from both platforms (TabNet and D-TISS), covering a 9-year period from 2015 to 2023, excluding 2024 due to incomplete DATASUS data for the period. The collected data included geographic region, the number of procedures performed, and in-hospital mortality rates. Statistics on in-hospital deaths were selected from the hospital mortality sections of TabNet and D-TISS. The data was grouped by geographic region and distributed over the years.

For data extraction within Public, the authors used a specific code assigned to this procedure established by the Public Procedure, Medicine, and OPM Management System (SIGTAP): 04.06.04.014–1. Data collection for the Private was obtained from the D-TISS Data Panel, published by ANS, and procedures were analyzed according to the Private coding system, which includes Vena Cava Filter (VCF) placement (code 30907080) and VCF implantation for pulmonary embolism prevention (code 40813240).

The steps for data collection, platform field selection, and Table adjustment were performed using the selenium-webdriver packages (v. 3.1.8, Selenium HQ, several collaborators worldwide) and pandas (v. 2.7.13, Lambda Foundry, Inc., and PyData Development Team, New York, USA). The Mozilla Firefox browser (v. 59.0.2, Mountain - California - USA) and geckodriver webdriver (v. 0.18.0, Mozilla Corporation, Bournemouth, England) were used. Following collection and treatment, all data were organized and grouped in a spreadsheet using Microsoft Office Excel 2016® (v. 16.0.4456.1003, Redmond - Washington - USA) software. These computer programs allowed automated content access (web scraping), the automated navigation codes were programmed in Python language (v. 2.7.13, Beaverton - Oregon - USA) using the Windows 10 Single Language operating system.

The examined variables included the total number of procedures, patient demographics (gender and age distribution), regional disparities in service provision, and associated mortality rates. For statistical analysis, generalized linear models with a Gamma distribution^19^ were performed to assess the relationship between the rate of procedures and average values with year, source of procedures (public or private), and region. For the death rate, a generalized linear model with a Poisson distribution was used, with the number of procedures as an offset. The results were presented as rate ratios, 95% Confidence Intervals, and p-values. The analyses were performed using R, version 4.1.1 (R Core Team. R: A Language and Environment for Statistical Computing. 2024. R version 4.4.1).

## Results

Between 2015 and 2023, a total of 21,630 CVF procedures were performed in Brazil, distributed between the Public and the Private. During this period, the Private system accounted for 57% (12,323) of all procedures, while the Public system accounted for 43% (9307). Notably, despite Private covering only 25% of the population, it performed a significantly higher proportion of CVF procedures (Table 1).^12^

**Table 1:** Absolute number of CVF procedures performed and incidence per 1 million beneficiaries in Public and Private health system between 2015 and 2023.

Year	n	Public Users	Incidence	n	Private Users	Incidence
2015	819	154,358,599	5.31	501	48,045,043	10.43
2016	864	157,552,513	5.48	885	46,319,412	19.11
2017	932	159,814,818	5.83	1195	45,396,739	26.32
2018	947	161,386,087	5.87	1202	45,142,951	26.63
2019	965	162,907,549	5.92	1413	44,992,550	31.41
2020	978	164,004,412	5.96	1493	45,160,477	33.06
2021	1234	163,553,169	7.54	1930	46,550,473	41.46
2022	1296	162,944,585	7.95	1832	47,918,398	38.23
2023	1272	162,943,737	7.81	1872	48,751,421	38.40
Average	1034	161,051,719	6.42	1369	46,475,274	29.45

Analyzing the incidence of CVF procedures per 1 million beneficiaries (Table 1), the private system exhibited a consistently higher incidence rate (average of 29.45 per 1 million beneficiaries) compared to the public system (average of 6.42). While the absolute number of procedures in the public system showed moderate growth (from 819 to 1272), the private system experienced a sharp increase (from 501 to 1872), peaking in 2021 (incidence: 41.46). Notably, the public system’s incidence rose during the pandemic (7.45 in 2021).

A predominance of women was observed in CVF procedures across both systems (Table 2). In Public, out of 9307, 59.15% were women (5506 cases) and 40.84% men (3801 cases). In Private (12,323), 56.25% were women (6932 cases) and 42.75% men (5268 cases). Overall, of 21,507 procedures with reported gender, 57.83% were women (12,438 cases) and 42.17% were men (9069 cases). Gender was unreported in only 123 cases (0.57%).

**Table 2:** Distribution by sex of CVF placement.

Sex	Public		Private		Total	
	n	%	n	%	n	%
Men	3801	40.84	5268	42.75	9069	41.93
Women	5506	59.15	6932	56.25	12,438	57.5
Not informed			123	1	123	0.57

**Fig. 1:**
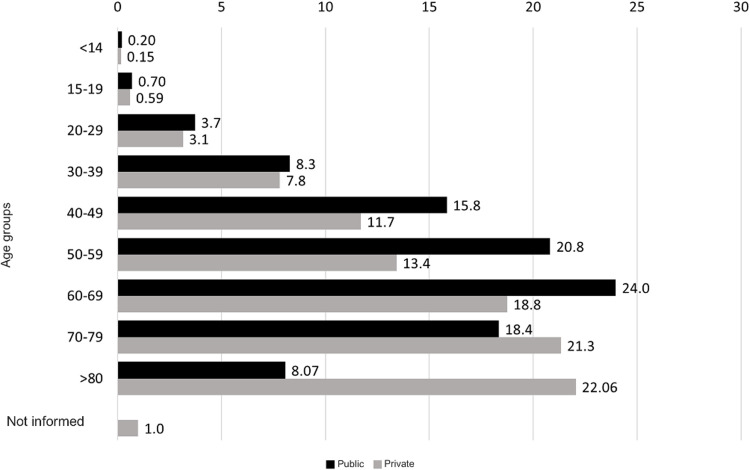
Distribution by age group of procedures carried out in Public and Private healthcare systems.

Fig. 1 presents the age distribution of procedures in Public and Private. In the Public system, the largest share (23.98%) fell in the 60‒69-year-old group. By contrast, in Private, the over-80-year-old cohort accounted for the highest proportion (2718 cases; 22.06%), versus 751 Public cases (8.07%). Additionally, 122 Private procedures (0.99%) lacked age data.

Table 3 presents the procedures performed by region. In Public, the Southeast led with 3771 procedures (40.518%), followed by the Northeast with 3074 (33.029%). In Private, the Southeast region again dominated with 8620 procedures (69.950%), then the Northeast with 1942 (15.759%), and the South with 1033 (8.383%).

**Table 3:** Procedures by region of the country.

Region	Public		Private		Total	
	n	%	n	%	n	%
North	128	1.38	163	1.32	291	1345
Northeast	3074	33.03	1.942	15.76	5016	23,190
Midwest	332	3.57	565	4.58	897	4147
Southeast	3771	40.52	8.620	69.95	12,391	57,286
South	2002	21.51	1033	8.38	3035	14,031
Total	9307	100.00	12,323	100.00	21,630	100,000

**Fig. 2:**
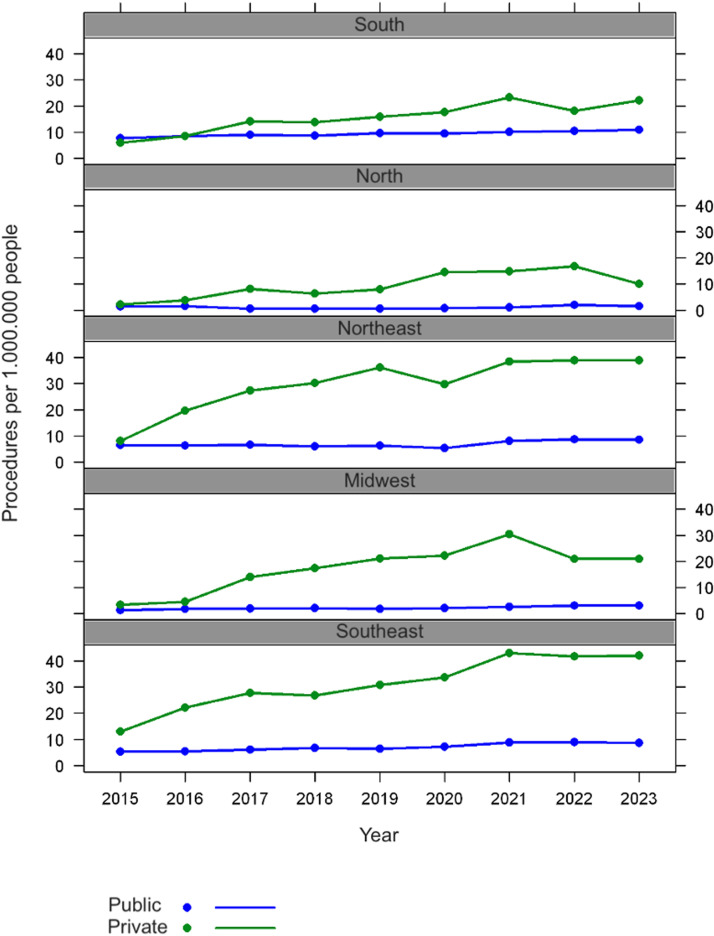
Procedures per 1.000.000 people.

Both systems show a predominance of procedures in the Southeast, substantial contributions from the Northeast and South, and minimal activity in the Midwest and North, a pattern consistent over time in the Brazil areas with the lowest demographic density (Fig. 2).

The adjusted model for the procedure rate, accounting for the region of origin, shows a significant change over the years (p-value < 0.001), with an estimated annual increase of 11% in the rate. Regarding the source of care, the procedure rate under Private is estimated to be 4.357 times higher than Public (p-value < 0.001) (Table 4).

**Table 4:** Procedure rate for 1.000.000 patients.

	Mean rate (95% CI)	p-value
**Year**	1.110 (1.074; 1.149)	<0.001
**Source**		
Public	Reference	
Private	4.357 (3.654; 5.193)	<0.001
**Region**		
Southeast	Reference	
Midwest	0.424 (0.325; 0.553)	<0.001
Northeast	0.971 (0.745; 1.267)	0.831
North	0.225 (0.172; 0.294)	<0.001
South	0.931 (0.712; 1.218)	0.600
Not adjusted by region		
**Source**		
Public	Reference	
Private	3.652 (2.883; 4.626)	<0.001

CI, Confidence Interval.

Analyzing the mortality associated with VCF implantation procedures (Table 5), overall rates were 7.4% in Private (918/12,323 procedures) versus 6.7% in Public (625/9307 procedures). In Public, regional mortality rates ranged from 3.1% in the North to 12.7% in the Midwest; in Private, from 3.7% in the North to 8.1% in the Northeast. The largest Public-Private disparity appeared in the Midwest (12.7% vs. 6.4%). In both the Southeast (7.0% vs. 7.4%) and South (6.7% vs. 7.4%) Private rates were slightly higher than Public.

**Table 5:** Absolute and relative distribution of deaths of patients undergoing surgical treatment per region for CVF placement in the SUS and PHS.

Region	Public			Private		
	Procedures	Deaths	Mortality rate	Procedures	Deaths	Mortality rate
North	128	4	3.1	163	6	3.7
Northeast	3074	181	5.9	1942	158	8.1
Midwest	332	42	12.7	565	36	6.4
Southeast	3771	264	7	8620	642	7.4
South	2002	134	6.7	1033	76	7.4
Total	9307	625	6.72	12,323	918	7.45

However, the adjusted model for the mortality rate showed no evidence of differences between healthcare sources (public vs. private, p-value = 0.066) and no significant change over the years (p-value = 0.053). A lower mortality rate in the North region compared to the reference region was noted (Table 6).

**Table 6:** Mortality rate per 1.000 procedures.

	Mean rate (95% CI)	p-value
**Year**	1.020 (1.001; 1.041)	0.056
**Source**		
Public	Reference	
Private	1.086 (0.975; 1.209)	0.134
**Region**		
Southeast	Reference	
Midwest	1.191 (0.937; 1.490)	0.139
Northeast	0.952 (0.836; 1.082)	0.453
North	0.474 (0.237; 0.835)	0.019
South	0.978 (0.836; 1.139)	0.779
Not adjusted by region		
**Source**		
Public	Reference	
Private	1.100 (0.994; 1.219)	0.066

CI, Confidence Interval.

## Discussion

This retrospective study utilizes population data from TabNet (DATASUS) and D-TISS (ANS) to assess trends in VCF use in Brazil. Rather than analyzing a sample, it covers a great part of the Brazilian population over nine years from 2015 to 2023. It is the first study to analyze the entire population of a continental developing country.

A total of 21,503 VCFs were placed during this period: 9307 in Public and 12,196 in Private. While Public averaged 6.42 procedures per 1 million beneficiaries, Private ‒ serving only 25% of the population ‒ had a fivefold higher rate (29.45 per 1 million). This contrasts with U.S. data for Medicare beneficiaries, which showed a decrease from 1578.0 to 641.0 per million (2010 and 2021)^20^ due to stricter guidelines - yet peak U.S. rates were still 20 times higher than Brazil's private system.

There is no nationwide epidemiological study to compare Brazilian rates with other Low-Middle Income Countries (LMIC), in India there is only one study from a tertiary referral center that indicates stricter use, adherent to absolute indications for usage in most cases, only 31 cases were analyzed.^21^ Market data from South Africa shows a trend in growing device use, baseline country-wide rates are unavailable.^22^ The absence of multi-center or population-level data from LMICs comparable to Brazil´s national registry limits cross-country analyses.

This disparity reflects more fragile health information systems and chronic funding constraints, consequences of deeper structural and systemic challenges that hinder the collection and consolidation of health data. This context places Brazil in a unique position, as the existence of its national registry, despite its own limitations, represents a rare source of data among its peer nations.

European data show intermediate usage: in 2012, the “Big Five” nations (France, Germany, Italy, Spain, and the UK) reported 9070 VCF placements or 28 per million.^23^ These contrasts reflect major differences in clinical practice across healthcare systems.

The fivefold disparity in VCF rate between Private and Public may reflect distinct scenarios: overutilization in the private sector, where financial incentives could drive unnecessary procedures, or unmet clinical need in the public system, where resource constraints may limit access to appropriate interventions, not seen in private where insured patients generally access endovascular procedures more easily, as high-resolution imaging and endovascular surgery are more available in private centers.^24^ Without indication-specific data, however, this critical distinction remains unresolved, highlighting the need for future studies.

VCF placement requires a team trained in endovascular surgery, predominantly available in private or private-affiliated hospitals. Within the public sector, high-complexity services are less available, particularly in the North and Northeast regions, limiting access to such procedures.^25^

Private healthcare systems typically operate under reimbursement models that may encourage more procedures. Although no direct evidence confirms financial drivers for VCFs in Private, fee-for-service models likely affect decisions, unlike the Public’s limited budget.^26^ Demographics in the Private population (older, more comorbid, higher socioeconomic status) might slightly influence VTE risk, but these factors alone do not explain the large gap in VCF usage.^27^^,^^28^

Recent trends in VCF placement show a clear contrast between the United States and Brazil. In the U.S., VCF use significantly declined after FDA alerts in 2010 and 2014, and the implementation of stricter guidelines aimed at correcting overuse and emphasizing filter retrieval, dropping among Medicare beneficiaries from 1578 to 641 insertions per 1 million between 2010 and 2021.^20^ Conversely, Brazilian data from 2015 to 2023 indicate a general upward trend in both the absolute number of procedures (Private + Public, from 1320 in 2015 to 3144 in 2023) and in rates per million inhabitants, particularly in the private healthcare sector, which rose from 10.18 to 38.34 per million, and also in Public, which increased from 5.31 to 7.81 per million, with a more pronounced increase from 2021 onwards.

Several hypotheses can explain these trends. Firstly, Brazil began with a considerably lower VCF usage baseline than the U.S., and its 140% increase over eight years might reflect an expansion of access to appropriate technology as its healthcare system evolves. Secondly, a rising incidence of VTE, possibly worsened by the COVID-19 pandemic and its known VTE risk, may also have contributed to the post-2021 surge.^29^

During the early COVID-19 pandemic (2020), the increased risk of VTE in critically ill patients led to some cases of early and increased use of VCF.^30^ As clinical experience grew (2021‒2022), therapeutic anticoagulation became established as the primary strategy, significantly reducing VTE incidence and mortality.^31^^,^^32^ Consequently, VCF use returned to traditional indications (anticoagulation failure or contraindications), leading to stabilization in utilization rates.^33^ Our findings align with this trend, showing peak usage in 2020 followed by stabilization higher level than the previous baseline.

The extent to which this increased usage represents overuse versus appropriate utilization remains uncertain, as specific implantation indications were not examined. Notably, implantation rates in Brazil's public healthcare system remained substantially lower than both U.S. and European benchmarks throughout the study period.^20^^,^^23^ While private sector implantation rates increased post-pandemic, they remained well below levels observed in systems with known overuse tendencies like the U.S. The slightly higher rates compared to European countries may reflect limited availability of recent European data rather than true overutilization.

A clear finding is that VCF procedures were more frequent in women, who accounted for 57.50% (*n* = 12,438) of all VCF placements, versus 41.93% (*n* = 9069) in men. This trend was observed in both healthcare settings: 59.16% in Public and 56.25% in Private. These consistent results suggest that the conditions requiring VCFs are more prevalent in women, independent of healthcare coverage.^10^^,^^34^

This finding may reflect an increased risk of PTE in women exposed to hormonal contraceptives, pregnancy, postpartum, or hormone replacement therapies.^10^^,^^20^

VCF placement is primarily age-associated, with approximately 74% of procedures in patients aged 50 or older. This trend is attributed to the higher risk of PTEdue to chronic conditions like cancer and kidney disease.^35^, ^36^, ^37^ Additionally, older individuals often experience mobility limitations, especially following major surgeries, and frequently use medications that affect coagulation, such as hormone replacement therapies and oncological treatments.^5^

Age group analysis reveals differences between Public and Private. In Public, procedures concentrate in ages 60‒69 (23.98%), and 50‒59 (20.82%). In Private, the distribution skews older, with 80+ (22.06%) and 70‒79 (21.34%) as the leading groups. The predominance of very advanced age groups (> 70 and particularly > 80-years) in the Private may suggest that individuals with higher socioeconomic status, who are more likely to have private health insurance and greater longevity, seek or have facilitated access to specific procedures within the private sector during this life stage. In contrast with Public patients witch frequently have a low-income status and may have reduced longevity.

An analysis of the regional distribution of healthcare procedures in Brazil reveals that the Southeast region accounts for most procedures, with 40.5% in the Public and 70% in the Private. This concentration can be attributed to the region's higher population density, widespread availability of supplementary health plans, and disproportionate share (51.9%) of the nation's vascular surgeons, representing one of the highest specialist densities per 100,000 inhabitants nationally.^28^ In contrast, the Midwest and North regions account for less than 5% of Public procedures and 6% of Private procedures, while containing only 3.0% and 9.7% of vascular surgeons, respectively (lowest per capita concentrations nationally). This highlights significant gaps in healthcare access, also reflecting demographic void. These disparities may lead to patients needing to travel interstate for complex cases and could necessitate the placement of VCFs.

The comparative analysis of in-hospital mortality following VCF placement in Brazil (2015‒2023) indicates that although the Private performed more procedures and consequently recorded a higher absolute number of deaths, its mortality rate was generally higher than that of the Public (7.42% vs. 6.63%). In 8- of 9-years analyzed, Private rates exceeded those of Public (2022 was the exception, and 2017 showed similar rates). Both systems exhibited fluctuations and peaks ‒ especially between 2020 and 2022 - likely influenced by the COVID-19 pandemic. However, these raw figures do not account for confounders such as case severity and complexity, differing eligibility criteria, and patient demographics. For example, Private patients may have access to more complex procedures or be transferred in a more critical condition, they may also have more comorbidities and cancer, while Public patients might face delayed access to care. Consequently, any inference about care quality requires a risk-adjusted analysis, which is currently unfeasible due to unavailable data. The lack of data on specific in-hospital mortality (VTE-related, procedure-related, or related to the underlying condition ‒ e.g., advanced malignancy, major trauma) remains unknown, further limiting the interpretability.

Overall, the in-hospital mortality rate for VCF placement is similar in both systems (6.7% in Public vs. 7.4% in Private). These rates largely reflect patients’ underlying severe conditions (e.g., VTE, cancer, or trauma), whereas mortality directly attribuTable to the VCF implantation procedure itself is usually below 1%,^8^^,^^38^ as shown in systematic reviews and clinical trials such as PREPIC.^19^^,^^39^ Direct comparisons across studies are complex due to variations in patient profiles, filter types, indications (therapeutic vs. prophylactic), and the definitions and follow-up periods for mortality. Regional variations observed in Brazil (Public rates ranging from 3.1% to 12.7% and Private rates from 3.7% to 8.1%) also suggest the influence of local factors and specific characteristics of each region’s population.

The mortality rates in Brazil between 2015 and 2023 find parallels in international studies for severe cohorts. In a large observational study in the USA (SAFE-IVC, with Medicare data from 2013‒2021) found a 30-day all-cause mortality rate of 14.6% in patients who received VCF; Brazil’s 6.7% and 7.4% rates are lower.^20^

### Limitations

This study has several limitations inherent to the use of aggregated administrative databases (DATASUS and D-TISS), including susceptibility to coding errors, reporting inconsistencies, and potential misclassification or underreporting. Data anonymization prevented longitudinal tracking, precluding identification of unique patients and hindering analyses of multiple procedures per individual (e.g., implantation, retrieval, reintervention). Inconsistent coding and the absence of patient-level variables (e.g., VTE severity, filter indication/type, comorbidities, socioeconomic status, complications) limited risk adjustment and assessment of evidence-based appropriateness. Furthermore, the lack of follow-up data made it impossible to evaluate long-term outcomes such as PE recurrence or retrieval rates. Age-stratified data for the private sector were unavailable, and out-of-pocket procedures, although likely infrequent, were not captured, introducing possible residual bias. Together, these limitations constrain our ability to assess adherence to clinical guidelines and evaluate long-term safety.

Despite this, the study offers a novel, comprehensive nationwide overview of VCF utilization in Brazil (2015–2023) across both public and private healthcare systems. By consolidating official data, it provides valuable real-world insights into differences in procedure volumes, thereby contributing to a better understanding of clinical practice and financing of this technology in the country. Looking ahead, future enhancements to the database, potentially including metrics such as filter retrieval rates and the specific clinical indications for implantation, could further strengthen the evidence base for guiding health policy in this field.

## Conclusions

In this analysis, based on real-world data from 2015 to 2023, a total of 21,630 VCF implantation procedures were performed in Brazil. This resulted in an average annual rate of 1.16 procedures per 10,000 inhabitants, considering the combined estimated populations covered by the Public and the Private.

The procedure rate per 10,000 inhabitants in the Private was 4.25 times higher than in the Public.

## Data availability statement

The datasets generated and/or analyzed during the current study are available from the corresponding author upon reasonable request.

## CRediT authorship contribution statement

**Clara Sanches Bueno:** Writing – original draft, Formal analysis. **Hedra Marques Santos:** Writing – original draft, Formal analysis. **Júlia Freire Castanheira:** Writing – original draft. **Bruno Jeronimo Ponte:** Data curation. **Felipe Soares Oliveira Portela:** Data curation. **Marcelo Fiorelli Alexandrino da Silva:** Writing – review & editing. **Marcelo Passos Teivelis:** Writing – review & editing. **Alexandre Fioranelli:** Writing – review & editing. **Nelson Wolosker:** Writing – review & editing, Supervision, Visualization.

## Conflicts of interest

The authors declare no conflicts of interest.

## References

[1] Takara N.C., da Costa Ferreira N., Murakami B.M., Lopes C.T. (2020). Development and validation of an informative manual on venous thromboembolism for the lay population. Einstein.

[2] Spencer F.A., Emery C., Lessard D. (2006). The worcester venous thromboembolism study. J Gen Intern Med.

[3] Heit J.A., Spencer F.A., White R.H. (2016). The epidemiology of venous thromboembolism. J Thromb Thrombolysis.

[4] Wendelboe A., Weitz J.I. (2024). Global health burden of venous thromboembolism. Arterioscler Thromb Vasc Biol.

[5] Konstantinides S.V., Meyer G., Becattini C. (2020). 2019 ESC guidelines for the diagnosis and management of acute pulmonary embolism developed in collaboration with the European Respiratory Society (ERS). Eur Heart J.

[6] Leiderman D.B.D., Zerati A.E., Vieira Mariz M.P., Wolosker N., Puech-Leão P., De Luccia N. (2019). The need for a Vena Cava filter in oncological patients with acute venous thrombosis: a marker of a worse prognosis. Ann Vasc Surg.

[7] Zerati A.E., Wolosker N., Yazbek G., Langer M., Nishinari K. (2005). Vena cava filters in cancer patients: experience with 50 patients. Clinics.

[8] Kaufman J., Kinney T., Streiff M. (2006). Guidelines for the use of retrievable and convertible vena cava filters: report from the Society of Interventional Radiology Multidisciplinary Consensus Conference. Surg Obes Relat Dis.

[9] Duffett L., Carrier M. (2017). Inferior vena cava filters. J Thromb Haemost.

[10] Leiderman D.B.D., Fiorelli M., Teivelis M.P., Stabellini N., Amaro Júnior E., Wolosker N. (2022). Temporal trends in vena cava filter implantation in public health system inpatients: an 11-year analysis of the largest city in Brazil. J Vasc Bras.

[11] Brasília: Ministry of Health; 2021. Brazil. Ministry of health. 20 Years of SUS. http://www.ccs.saude.gov.br/sus20anos/mostra/pdf/painel5.pdf. Portuguese.

[12] Brasília: ANS; 2021. National Supplementary Health Agency (Brazil). Sector profile: data and indicators. http://www.ans.gov.br/perfil-do-setor/dados-e-indicadores-do-setor/sala-de-situacao. Portuguese.

[13] Andrade A.O., de Jesus S.R., Mistro S. (2023). Hospitalizações no Brasil pelas estimativas da Pesquisa Nacional de Saúde, 2013 e 2019. Rev Saude Publ.

[14] Saeed M.J., Turner T.E., Brown D.L. (2017). Trends in inferior vena cava filter placement by indication in the United States From 2005 to 2014. JAMA Intern Med.

[15] Olanipekun T., Ritchie C., Abe T. (2024). Updated trends in inferior vena cava filter use by indication in the United States after food and drug administration safety warnings: a decade analysis from 2010 to 2019. J Endovasc Ther.

[16] Louzada A.C.S., Diamante Leiderman D.B., Alexandrino da Silva M.F. (2024). Epidemiology of the use of inferior vena cava filters in Brazil between 2008 and 2019. Vascular.

[17] Bueno C., Santos H., Castanheira J., et al. Epidemiological analysis of all vena cava filter placement over 9 years in Brazil: trends and mortality rates in a population of over 200 million. MedRix. 2025;2025.07.07.25331036.10.1016/j.clinsp.2026.10087441722447

[18] Strengthening the reporting of observational studies in epidemiology. https://www.strobe-statement.org/checklists/. 2025. STROBE checklist.

[19] Paula G.A. (2004).

[20] Ferro E.G., Mackel J.B., Kramer R.D. (2024). Postmarketing surveillance of inferior vena cava filters among US medicare beneficiaries. JAMA.

[21] Kumar S., Kumar A., Kumar M. (2021). Clinical profile and outcomes of COVID-19 in haematological malignancies: experience from tertiary care centre in India. Indian Heart J.

[22] Medical Device Network. Market share analysis: inferior vena cava filters in South Africa [Internet]. 2023 [cited 2025 Aug 8]. Available from: https://www.medicaldevice-network.com/data-insights/market-share-analysis-inferior-vena-cava-filters-south-africa/?cf-view.

[23] Wang S.L., Lloyd A.J. (2013). Clinical review: inferior vena cava filters in the age of patient-centered outcomes. Ann Med.

[24] Franco R.P., Chula D.C., de Moraes T.P., Campos R.P. (2022). Health insurance provider and endovascular treatment availability are associated with different hemodialysis vascular access profiles: a Brazilian national survey. Front Nephrol.

[25] da Silva Barbosa R., Fagnani E. (2021). The regionalization process for universal health coverage in Brazil (2008-2015). Healthcare.

[26] Dowd B.E., Laugesen M.J. (2020). Fee-for-service payment is not the (main) problem. Health Serv Res.

[27] Lutsey P.L., Zakai N.A. (2023). Epidemiology and prevention of venous thromboembolism. Nat Rev Cardiol.

[28] Paim J., Travassos C., Almeida C., Bahia L., Macinko J. (2011). The Brazilian health system: history, advances, and challenges. Lancet.

[29] Boonyawat K., Chantrathammachart P., Numthavaj P. (2020). Incidence of thromboembolism in patients with COVID-19: a systematic review and meta-analysis. Thromb J.

[30] Hadid T., Kafri Z., Al-Katib A. (2021). Coagulation and anticoagulation in COVID-19. Blood Rev.

[31] Georgiadis G.S., Argyriou C., Tottas S., Foutzitzi S., Drosos G. (2022). Extensive inferior vena cava thrombosis related to COVID-19 infection in a patient with retrievable filter due to multiple pelvic bone fractures. Vasc Spec Int.

[32] Sobreira M., Marques M., Paschoa A. (2024). Guidelines on deep vein thrombosis of the Brazilian Society of Angiology and Vascular Surgery. J Vasc Bras..

[33] Cena T., Bazzano S., Berni P. (2020). Inferior vena cava filter in a patient with COVID-19 pneumonia to prevent a massive pulmonary embolism. Ann Vasc Surg.

[34] Scheres L.J.J., van Hylckama Vlieg A., Cannegieter S.C. (2022). Sex-specific aspects of venous thromboembolism: what is new and what is next?. Res Pract Thromb Haemost.

[35] Poenou G., Dumitru Dumitru T., Lafaie L. (2022). Pulmonary embolism in the cancer associated thrombosis landscape. J Clin Med.

[36] Singh J., Khadka S., Solanki D. (2021). Pulmonary embolism in chronic kidney disease and end-stage renal disease hospitalizations: trends, outcomes, and predictors of mortality in the United States. SAGE Open Med.

[37] Rodrigues A.C.T., Cordovil A., Mônaco C.G. (2013). Assessing prognosis of pulmonary embolism using tissue-Doppler echocardiography and brain natriuretic peptide. Einstein.

[38] Decousus H., Leizorovicz A., Parent F. (1998). A clinical trial of vena caval filters in the prevention of pulmonary embolism in patients with proximal deep-vein thrombosis. N Engl J Med.

[39] PREPIC study group (2005). Eight-year follow-up of patients with permanent vena cava filters in the prevention of pulmonary embolism: the PREPIC randomized study. Circulation.

